# Inherited Polymorphisms in Hyaluronan Synthase 1 Predict Risk of Systemic B-Cell Malignancies but Not of Breast Cancer

**DOI:** 10.1371/journal.pone.0100691

**Published:** 2014-06-20

**Authors:** Hemalatha Kuppusamy, Helga M. Ogmundsdottir, Eva Baigorri, Amanda Warkentin, Hlif Steingrimsdottir, Vilhelmina Haraldsdottir, Michael J. Mant, John Mackey, James B. Johnston, Sophia Adamia, Andrew R. Belch, Linda M. Pilarski

**Affiliations:** 1 University of Alberta and Cross Cancer Institute, Edmonton, Alberta, Canada; 2 University of Iceland, Reykjavik, Iceland; 3 Landspitali University Hospital, Reykjavik, Iceland; 4 Dept. of Hematology, Cancer Care Manitoba and the University of Manitoba, Winnipeg, Manitoba, Canada; 5 Medical Oncology, Dana-Farber Cancer Institute, Harvard Medical School, Boston, Massachusetts, United States of America; University of Saarland Medical School, Germany

## Abstract

Genetic variations in the hyaluronan synthase 1 gene (HAS1) influence HAS1 aberrant splicing. HAS1 is aberrantly spliced in malignant cells from multiple myeloma (MM) and Waldenstrom macroglobulinemia (WM), but not in their counterparts from healthy donors. The presence of aberrant HAS1 splice variants predicts for poor survival in multiple myeloma (MM). We evaluated the influence of inherited HAS1 single nucleotide polymorphisms (SNP) on the risk of having a systemic B cell malignancy in 1414 individuals compromising 832 patients and 582 healthy controls, including familial analysis of an Icelandic kindred. We sequenced *HAS1* gene segments from 181 patients with MM, 98 with monoclonal gammopathy of undetermined significance (MGUS), 72 with Waldenstrom macroglobulinemia (WM), 169 with chronic lymphocytic leukemia (CLL), as well as 34 members of a monoclonal gammopathy-prone Icelandic family, 212 age-matched healthy donors and a case-control cohort of 295 breast cancer patients with 353 healthy controls. Three linked single nucleotide polymorphisms (SNP) in *HAS1* intron3 are significantly associated with B-cell malignancies (range p = 0.007 to p = 10^−5^), but not MGUS or breast cancer, and predict risk in a 34 member Icelandic family (p = 0.005, Odds Ratio = 5.8 (OR)), a relatively homogeneous cohort. In contrast, exon3 SNPs were not significantly different among the study groups. Pooled analyses showed a strong association between the linked *HAS1* intron3 SNPs and B-cell malignancies (OR = 1.78), but not for sporadic MGUS or for breast cancer (OR<1.0). The minor allele genotypes of HAS1 SNPs are significantly more frequent in MM, WM, CLL and in affected members of a monoclonal gammopathy-prone family than they are in breast cancer, sporadic MGUS or healthy donors. These inherited changes may increase the risk for systemic B-cell malignancies but not for solid tumors.

## Introduction

Hyaluronan synthase 1 (HAS1) produces hyaluronan (HA), a polysaccharide with complex biological effects [1]. Here, we evaluated the influence of inherited HAS1 single nucleotide polymorphisms (SNP) on the risk of having a systemic B cell malignancy in 832 patients and 582 controls. Using targeted sequencing of HAS1 SNPs to unequivocally genotype patient populations and to identify potential low penetrance mutations, we reduced the uncertainties associated with array screening and genome-wide association studies. Herein, we show that three HAS1 intron3 SNPs (rs11084110, rs11084109 and rs11669079) are significantly more frequent in patients with systemic B-cell malignancies or in members of a four-generation Icelandic kindred affected by a monoclonal gammopathy-prone phenotype [2]; [3] than in healthy donors, unaffected Icelandic family members or patients with solid tumors. Overall, the study of 1414 subjects, including 832 patients and 582 healthy controls, suggests that these inherited changes may predispose to the development of systemic B cell malignancies (multiple myeloma, chronic lymphocytic leukemia or Waldenstrom macroglobulinemia), but do not predispose to sporadic monoclonal gammopathy of undetermined significance (MGUS) or breast cancer.

Hyaluronan synthase 1 products, encoded by the *HAS1* gene, appear central to the events giving rise to B cell malignancies and to disease progression [1]. HAS1 is aberrantly spliced in malignant cells from multiple myeloma (MM) and Waldenstrom macroglobulinemia (WM), but not in their counterparts from healthy donors [4]. HA produced by HAS1 splice variants is likely to contribute to mitotic abnormalities and to malignant spread/migration [1]; [4]; [5], suggesting that aberrant splicing may be an important contributor to systemic B cell malignancies. The presence of the aberrant HAS1Vb intronic splice variant correlated with poor survival in MM patients [4]. In transfectants, a HAS1 variant is oncogenic *in vivo* and *in vitro* [6], possibly resulting from induction of chromosomal instability by aberrant intracellular HA and promotion of malignant spread by extracellular HA [1]. Inherited single nucleotide polymorphisms (SNPs) and mutational hotspots in HAS1 exon3 through intron3 influence HAS1 pre-mRNA splicing [7]; [8]. A major strength of this study is that we have analyzed the *HAS1* polymorphisms in five different disease cohorts and two geographically independent populations (western Canadians and the Icelandic kindred) with their appropriate healthy donor control groups. This is the first report that risk for B-cell malignancies and an inherited monoclonal gammopathy-prone phenotype is directly correlated with intronic *HAS1* SNPs, likely by promoting aberrant HAS1 splicing.

## Methods

### Study Subjects

Patients with MM, WM or sporadic MGUS were seen at the Cross Cancer Institute and the University of Alberta hospital (Edmonton, AB), and diagnosed according to recommended guidelines [9]; [10] CLL cells were obtained from the Manitoba CLL Tumor Bank at the Manitoba Institute of Cell Biology. This study was approved by Ethics Review Boards from the University of Alberta, the University of Manitoba, the University of Iceland and Alberta Health Services. Subjects provided written informed consent. Approval for the familial studies was from the Icelandic National Bioethics Committee, license number 8-107-S1, Data Protection Authority: 20096080676. We sequenced DNA from 1,414 individuals (Table 1): this includes a total of 582 healthy control subjects. Controls for B-cell malignancies were 212 anonymous age-matched donors. For breast cancer, 295 anonymous blood samples from patients and 353 matched healthy controls were from the Alberta Research Tumor Bank. For all groups, peripheral blood was taken at the time of diagnosis or at follow-up. We genotyped PB from 34 members from four generations of a monoclonal gammopathy-prone Icelandic family [2]; [3]. Family members were of both genders; “controls” were the unaffected family members. Affected family members were those with MGUS, MM, WM or a hyper-Ig phenotype. Both the Icelandic kindred and the patients from western Canada are predominantly of Caucasian descent.

**Table 1:** Characteristics of study participants (N = 1414).

Study Group	No of samples	Age Range	Sample type
MGUS	98	44–87 (n = 67)	PB
MM	181	46–91 (n = 66)	PB
CLL	169	37–82 (n = 66)	Purified malignant B cells
WM	72	50–82 (n = 66)	PB
Controls	212	Above 60	PB
Icelandic kindred	34	4 generations	PB
Breast cancer cases	295	29–80	PB
Breast cancer Controls	353	29–80	

### Genotyping and Analysis

Peripheral blood mononuclear cells were isolated and genomic DNA was purified from 5×10^6^ PBMCs using QIAamp DNA Blood mini kit (QIAGEN). For two Icelandic samples, DNA was isolated from paraffin-embedded tissue, and amplified with whole genome amplification (WGA). Primers were designed based on the consensus human *HAS1* NCBI gene coding sequence (NCBI Reference Sequence: NC_000019.10) (Table S1 in File S1). A *HAS1* gene segment from exon3 to intron3 inclusive was amplified in a 50 µl PCR reaction mix (Table S2 in File S1).

### Quality Control

To ensure the genotyping results were accurate, we randomly re-sequenced selected DNA samples. For high fidelity *Taq* DNA polymerase, an enzyme lot was used with an error rate confirmed by us to be 3.0×10^−4^ error/bp. DNA sequencing profiles were analyzed with SeqScape V2.1 for base-calling software/alignment (Applied Biosystems (ABI), CA), confirmed manually by visual inspection of the sequencing profile.

### Subcloning and Direct Sequencing

PCR-amplified gene segments were sequenced using two methods: 1) by subcloning in an appropriate vector or 2) by direct sequencing of double or single stranded PCR products. For each patient whose PCR products were subcloned, a minimum of 8 subclones were sequenced in both directions. If a given base was present in all subclones, the two alleles were considered to be homozygous. Heterozygosity was defined as having at least 1/8 subclones with the second allele. 40% of samples were sequenced using plasmid subcloning and 60% were sequenced directly using Big-Dye 3.1 (ABI) on the ABI3130xl DNA analysis system. Obtained sequences were compared to the NCBI *HAS1* reference sequence (NC_000019.10).

### Statistical Analysis

Hardy-Weinberg equilibrium (HWE) tests were performed for each SNP. We assessed for deviations from HWE using the chi-square test, with 5 degrees of freedom. The SNPs were only considered for further analysis if the allele frequency conformed to HWE expectation. This ensures that any loss of heterozygosity that might be present in malignant cells is excluded from analysis [11]. The difference in genotype and allele frequencies between patients and healthy controls were analyzed by the Fischer exact test: probability values (P values) of <0.05 were considered statistically significant. The *HAS1* alleles designated as “minor” by NCBI are the most frequent in the populations studied, with the exception of the Icelandic kindred, but we followed the accepted NCBI dbSNP database convention for naming.

Unconditional logistic regression analysis was performed to obtain odds ratios (OR) and 95% confidence interval (CI) to independently estimate relative risks for each SNP, using Statistical Analysis Software (SAS institute). The independent cohorts were then combined for Forest plot analysis without any weightage. They show the amount of variation between the studies and an estimate of the overall result. Linkage disequilibrium analysis was done using *Haploview* bioinformatic software (MIT/Harvard Broad Institute). Linked SNPs (rs11084110, rs11084109 and rs11669079) show strong LD patterns in all the four case groups and D’ values are above 90. In contrast, the exon3 SNPs (rs61736495, rs11084111) are in equilibrium; only rs11084111 was present in the Icelandic kindred.

## Results

### *HAS1* Polymorphisms

**Figure 1:**
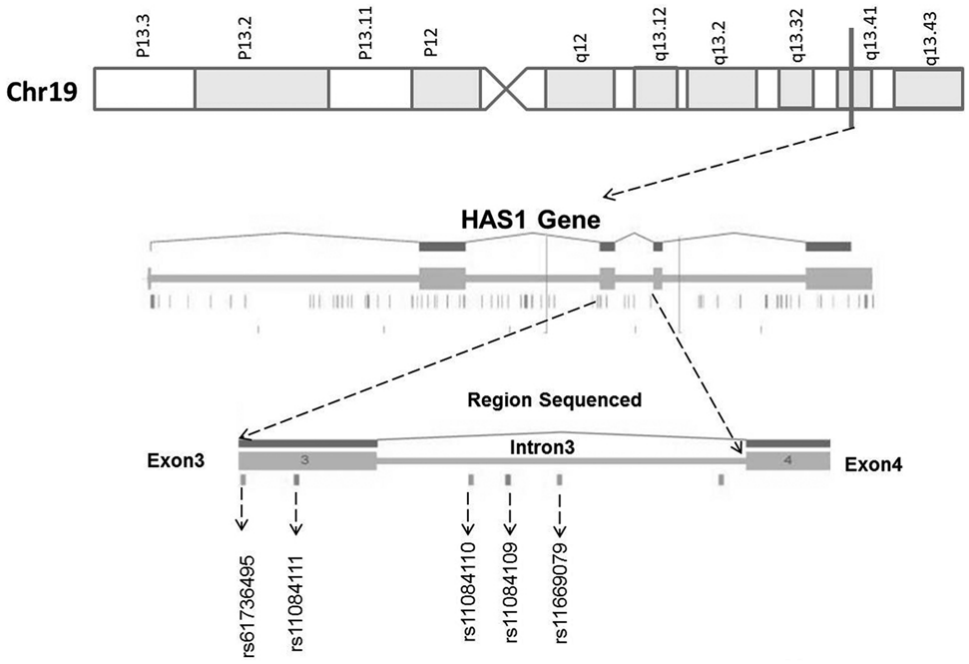
A schematic diagram of HAS1 gene locus in chromosome 19q13. Some parts of Figure 1 were created using Gene Window Software http://genewindow.nci.nih.gov/.

HAS1 gene segments from 1414 individuals were sequenced (Table 1). In the dbSNP database, ten *HAS1* SNPs occur in the region sequenced (Figure 1, Table-S3 in File S1); of these only five met the Hardy-Weinberg Equilibrium criterion of p>0.05; these were two SNPs in exon 3 and three SNPs in intron 3.

### HAS1 Genotype and Minor Allele Frequencies in an Icelandic Kindred

We analyzed 34 members of an Icelandic family predisposed to monoclonal gammopathies, including IgM gammopathy, CLL, MM and WM [2]; [3] (Table 2). Twelve family members showed increased production of immunoglobulin *in vitro*, referred to as hyper-responders [3]. The 12 hyper-responders plus two family members diagnosed MGUS, two with WM and one with MM (a total of 17 family members) were termed “affected” family members. Controls were 17 unaffected family members who lacked any evidence of monoclonal gammopathies and did not have a hyper-responder phenotype. In the Icelandic kindred, the frequencies of the linked intron3 SNPs (rs11084110, rs11084109 and rs11669079) for affected members were significantly different from those of unaffected members (genotype frequencies p = 0.007 for the intron3 SNPs). In contrast, the exon3 SNP rs11084111 is not significantly different between affected and unaffected family members, providing an internal control. The minor allele frequency for the linked intron3 SNPs was 0.74 for affected and 0.32 for unaffected family members (Table 2), a highly significant difference (p = 0.0005).

**Table 2:** Genotype and Allele frequencies of *HAS1* SNPs in Icelandic kindred*.

*HAS1* SNP	Status		Genotype frequency			Minor allele frequency		
		Major allele Homozygous	Heterozygous	Minor allele Homozygous	P	Major Allele	Minor Allele	P
rs11084111	Affected	15/17 (88%)	2/17 (12%)	0/17 (0%)	0.36	32 (0.94)	2 (0.06)	0.38
	Unaffected	13/17 (76%)	4/17 (14%)	0/17 (0%)		30 (0.88)	4 (0.12)	
rs11084110	Affected	1/17 (6%)	7/17 (41%)	9/17 (53%)	0.007	9 (0.26)	25 (0.74)	0.0005
	Unaffected	9/17 (53%)	5/17 (29%)	3/17 (18%)		23 (0.68)	11 (0.32)	
rs11084109	Affected	1/17 (6%)	7/17 (41%)	9/17 (53%)	0.007	9 (0.26)	25 (0.74)	0.0005
	Unaffected	9/17 (53%)	5/17 (29%)	3/17 (18%)		23 (0.68)	11 (0.32)	
rs11669079	Affected	1/17 (6%)	7/17 (41%)	9/17 (53%)	0.007	9 (0.26)	25 (0.74)	0.0005
	Unaffected	9/17 (53%)	5/17 (29%)	3/17 18%)		23 (0.68)	11 (0.32)	

*P values are calculated using fisher exact test, allele frequencies are shown in parentheses.

### Minor Allele Frequencies (MAF) for Study Cohorts

The allele frequencies for patients and their respective control groups were determined (Table 3). For *HAS1* exon3 SNPs the allele distribution between the cancer groups and their controls are not significantly different. In contrast however, the linked *HAS1* intron3 SNPs, the MAF are significantly higher in CLL and WM than they are in the age-matched control group. For all of the intron3 SNPs, the MAF is 0.80 for CLL and 0.81 for WM compared to 0.75 for MM and 0.63 for sporadic MGUS. For MM, sporadic MGUS and the breast cancer group (0.71), the MAFs for the linked *HAS1* intron3 SNPs are not significantly different from their respective control groups. For *HAS1* exon3 SNPs the allele distribution between the cancer groups and their controls are not significantly different.

**Table 3:** Allele frequencies of *HAS1* SNPs in Case cohorts (MGUS, MM, CLL and WM)*.

SNPs	MGUS Cohort			MM Cohort			CLL Cohort			WM Cohort		
	Major Allele	Minor Allele	P	Major Allele	Minor	P	Major Allele	Minor Allele	P	Major Allele	Minor	P
rs61736495												
Case	194 (0.99)	2 (0.01)	0.152	355 (0.98)	7 (0.902)	0.414	329 (0.97)	9 (0.03)	0.88	143 (0.99)	1 (0.01)	0.138
Control	412 (0.97)	12 (0.03)		412 (0.97)	12 (0.03)		412 (0.97)	12 (0.03)		412 (0.97)	12 (0.03)	
rs11084111												
Case	179 (0.91)	17 (0.09)	0.125	329 (0.91)	33 (0.09)	0.04	313(0.93)	25 (0.07)	0.265	132 (0.92)	12 (0.08)	0.204
Control	401 (0.95)	23(0.05)		401 (0.95)	23(0.05)		401 (0.95)	23(0.05)		401 (0.95)	23(0.05)	
rs11084110												
Case	72 (0.37)	126(0.63)	0.44	89(0.25)	273 (0.75)	0.007	68 (0.20)	270 (0.80)	5.0×*^10-5^*	28(0.19)	116 (0.81)	5.0×*^10-4^*
Control	141 (0.33)	283 (0.67)		141 (0.33)	283 (0.67)		141 (0.33)	283 (0.67)		141 (0.33)	283 (0.67)	
rs11084109												
Case	73 (0.37)	125(0.63)	0.44	90 (0.25)	272(0.75)	0.006	68 (0.20)	270 (0.80)	3.0×*^10-7^*	28(0.19)	116 (0.81)	*3.7×^10-4^*
Control	143 (0.34)	281 (0.66)		143 (0.34)	281 (0.66)		143 (0.34)	281 (0.66)		143 (0.34)	281 (0.66)	
rs11669079												
Case	72 (0.37)	126(0.63)	0.44	89(0.25)	273 (0.75)	0.007	68 (0.20)	270 (0.80)	5.0×*^10-5^*	28(0.19)	116 (0.81)	5.0×*^10-4^*
Control	141 (0.33)	283 (0.67)		141 (0.33)	283 (0.67)		141 (0.33)	283 (0.67)		141 (0.33)	283 (0.67)	
												

*P values are calculated using fisher exact test, allele frequencies are shown in parentheses. Alleles are designated as major or minor based on the NCBI database.

### Association between *HAS1* SNP Genotypes and B-cell Malignancies

Two-tailed unconditional logistic regression was used to determine the effect of *HAS1* SNP genotypes on the presumptive risk of B-cell malignancy (Table 4). No assumptions were made about the effect of the SNPs prior to analysis. Odds ratios (OR) measure the impact of a given *HAS1* SNP on the risk of having a B-cell malignancy (Table 4). Each cancer group was compared to its control group.

**Table 4:** HASI SNP genotype associates with risk of B-cell malignancy*.

SNP	Genotype	Controls	MGUS	OR (95% CI) P	MM	OR (95% CI) P	CLL	OR (95% CI) P	WM	OR (95% CI) P
rs61736495	GG	200	96	1. 00	174	1. 00	160	1. 00	71	1. 00
*HAS1*	GA	12	2	0.35 (0.08–1.58) 0.172	7	0.67 (0.25–1.74) 0.4	9	0.93 (0.40–2.25) 0.88	1	0.24 (0.03–1.86) 0.16
	AA	0	0	–	0	–	0	–	0	–
	OR_trend_			0.34 (0.08–1.59) 0.177		0.67 (0.26–1.73) 0.4		0.93 (0.40–2.28) 0.88		0.24 (0.03–1.83) 0.16
rs11084111	CC	189	81	1. 00	151	1. 00	145	1. 00	60	1. 00
*HAS1*	CT	23	17	1.72 (0.90–3.40) 0.116	27	1.46 (0.81–2.66) 0.2	23	1.30 (0.70–2.40) 0.4	12	1.64 (0.77–3.50) 0.19
	TT	0	0	–	3	–	1	–	0	–
	OR_trend_			1.65 (0.86–3.17) 0.129		1.43 (1.00–3.03) 0.05		1.39 (0.70–2.40) 0.26		1.60 (0.76–3.27) 0.21
rs11084110	GG	21	17	1. 00	14	1. 00	8	1. 00	2	1. 00
*HAS1*	GA	99	38	0.47 (0.22–0.99) 0.05	61	0.92 (0.43–1.95) 0.84	52	1.37 (0.60–3.32) 0.47	24	2.54 (0.55–11.6) 0.22
	AA	92	43	0.57 (0.30–1.20) 0.14	106	1.72 (0.83–3.59) 0.14	109	3.11 (1.37–7.35) 0.01	46	5.25 (1.18–23.3) 0.02
	OR_trend_			0.85 (0.60–1.22) 0.39		1.52 (1.14–2.09) **0.008**		2.(1.41–2.76))**1.0×10^−5^**		2.10 (1.33–3.26) **0.002**
rs11084109	GG	21	18	1. 00	14	1. 00	8	1. 00	2	1. 00
*HAS1*	GA	101	37	0.42 (0.20–0.90) 0.02	62	0.92 (0.43–1.94) 0.82	52	1.37 (0.56–3.25) 0.5	24	2.5 (0.50–11.3) 0.23
	AA	90	43	0.55 (0.30–1.115) 0.11	105	1.75 (0.84–3.64) 0.13	109	3.17 (1.34–7.51) 0.008	46	5.36 (1.20–23.9) 0.02
	OR_trend_			0.85 (0.60–1.22) 0.39		1.53 (1.12–2.10) **0.007**		2 (1.41–2.82))**1.0×10^−5^**		2.10(1.33–3.30) **0.001**
rs11669079	TT	21	17	1. 00	14	1. 00	8	1. 00	2	1. 00
*HAS1*	TA	99	38	0.47 (0.22–0.99) 0.05	61	0.92 (0.43–1.95) 0.84	52	1.37 (0.60–3.32) 0.47	24	2.54 (0.55–11.6) 0.22
	AA	92	43	0.57 (0.30–1.20) 0.14	106	1.72 (0.83–3.59) 0.14	109	3.11 (1.37–7.35) 0.01	46	5.25 (1.18–23.3) 0.02
	OR_trend_			0.85 (0.60–1.22) 0.39		1.52 (1.14–2.09) **0.008**		2 (1.41–2.76) **1.0×10^−5^**		2.10 (1.33–3.26) **0.002**

*All comparisons are of case subjects with control subjects. All groups met Hardy Weinberg Equilibrium. Allelic odds ratios and the corresponding 95% confidence intervals were calculated for the association analysis and are shown in the table. CI denotes confidence interval, OR denotes odds ratio. The genome position is based on the National Centre for Biotechnology Information database. For CLL samples, a cohort of 20 patients provided both enriched B cells and buccal cells; both populations gave identical results. No assumptions were made about the effect of the SNPs prior to analysis.

As compared to the healthy controls, for the linked *HAS1* intron3 SNPs, the genotypes having the “minor” allele were each associated with an increased risk of B-cell malignancy but not of MGUS, perhaps because only 1–2% of sporadic MGUS transform to MM. For the exon 3 SNPs, there was no association between *HAS1* SNP genotype and B-cell malignancies or MGUS. The intronic *HAS1* SNP rs11084110 was associated with 1.5-fold increase in the risk for MM (OR = 1.53; p = 0.008). The OR was 2.0 for CLL and 2.1 for WM, both of which have a higher familial incidence than does MM. Similar results were observed for the other two intronic SNPs (Table 4). To determine whether or not the linkage between intronic HAS1 SNPs and risk was restricted to B cell malignancies, we also evaluated a cohort of breast cancer patients. There was no difference between breast cancer patients and their matched healthy control group, indicating a lack of association with HAS1 SNPs (Table 5). Comparison of the B-cell cancer group (MM, CLL and WM) with the breast cancer subjects shows that for all the three linked SNPs, the systemic B cell malignances have a significantly greater association than does breast cancer (p≥0.01). Thus, the solid tumor group and its controls provide a negative control for the patient groups harboring systemic B-cell malignancies. Our findings showed that the linked *HAS1* intron3 SNPs, had a significant association with all B-cell malignancies studied (Table 4), an association that appears restricted to B-cell malignancies.

**Table 5:** *HAS*1 SNPs do not associate with breast cancer or ductal carcinoma in situ (DCIS).

SNP	Genotype	Controls	Breast Cancer	OR (95% CI) P	Subset DCIS	OR (95% CI) P	Breast Cancer pooled	OR (95% CI) P
rs61736495	GG	352	237	1. 00	55	1. 00	292	1. 00
*HAS1*	GA	0	2	–	1	–	3	–
	AA	0	0	–	0	–	0	–
	OR_trend_			–		–		–
rs11084111	CC	339	224	1. 00	53	1. 00	277	1. 00
*HAS1*	CT	13	15	1.74 (0.81–3.74) 0.15	3	1.47(0.40–5.35) 0.55	18	1.69 (0.81–3.51) 0.15
	TT	0	0	–	0	–	0	–
	OR_trend_			1.72 (0.81–3.65) 0.15		1.47(0.40–5.21) 0.55		1.67 (0.81–3.44) 0.16
rs11084110	GG	26	15	1. 00	4	1. 00	19	1. 00
*HAS1*	GA	144	104	1.25 (0.63–2.48) 0.52	32	1.44 (0.47–4.42) 0.52	136	1.29 (0.68–2.44) 0.42
	AA	182	120	1.14 (0.58–2.24) 0.69	20	0.71(0.22–2.25) 0.56	140	0.98 (0.52–1.85) 0.96
	OR_trend_			0.99 (0.76–1.28) 0.94		0.69 (0.45–1.05) 0.08		0.88 (0.69–1.12) 0.31
rs11084109	GG	26	15	1. 00	4	1. 00	19	1. 00
*HAS1*	GA	144	104	1.25 (0.63–2.48) 0.52	32	1.44 (0.47–4.42) 0.52	136	1.29 (0.68–2.44) 0.42
	AA	182	120	1.14 (0.58–2.24) 0.69	20	0.71(0.22–2.25) 0.56	140	0.98 (0.52–1.85) 0.96
	OR_trend_			0.99 (0.76–1.28) 0.94		0.69 (0.45–1.05) 0.08		0.88 (0.69–1.12) 0.31
rs11669079	TT	26	15	1. 00	4	1. 00	19	1. 00
*HAS1*	TA	144	104	1.25 (0.63–2.48) 0.52	32	1.44 (0.47–4.42) 0.52	136	1.29 (0.68–2.44) 0.42
	AA	182	120	1.14 (0.58–2.24) 0.69	20	0.71(0.22–2.25) 0.56	140	0.98 (0.52–1.85) 0.96
	OR_trend_			0.99 (0.76–1.28) 0.94		0.69 (0.45–1.05) 0.08		0.88 (0.69–1.12) 0.31

### Pooled Association Analysis

**Figure 2:**
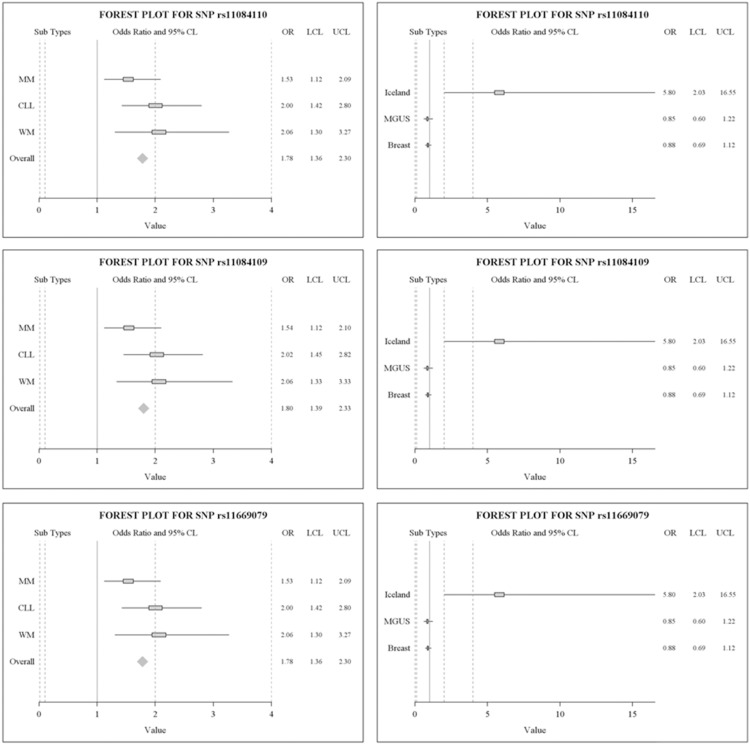
Forest plots of three linked SNPs in B-cell Malignancies, the Icelandic kindred, breast cancer and MGUS. The left column of three panels show the odds ratios of B cell malignancy for the three linked intronic *HAS1* SNPs. The OR denotes the odds of having the minor allele as assessed by genotype. The X-axis corresponds to odds ratio. The horizontal line represents 95% confidence interval. Each box represents the OR point estimate. The diamond represents the odds ratios obtained from pooled analysis with 95% confidence interval. Each group is compared to the appropriate control group. The right column of three panels show odds ratios for the Icelandic kindred, MGUS and breast cancer; each individual group is compared to its own control group as indicated in results. The cumulative risks were not estimated for these last three groups as they are unrelated to each other. These plots show that there were no associations of risk with the three linked SNPs for sporadic MGUS or breast cancer. There was a very strong association of the minor allele with risk for the B cell malignancies (pooled OR = 1.78) and the Icelandic Kindred (OR = 5.8). Note difference in scale values between panels in the two columns.

For increased power to demonstrate a relationship between *HAS1* SNPs and the risk of a B-cell malignancy, three case cohorts (MM, CLL and WM) were pooled for analysis (Figure 2). When MM, CLL and WM were evaluated as a single group in comparison with the healthy control group, we found a significant association between the linked *HAS1* intron3 SNPs, and the risk of systemic B-cell malignancy. For the three *HAS1* intronic SNPs (OR = 1.78–1.80; p<0.001), the overall risk was 1.78 (Figure 2, left column of panels) for the group of B cell malignancies. For the Icelandic family members the overall risk was 5.8; no such risk was seen for breast cancer or for the cohort of patients with sporadic MGUS (OR = 0.85 or 0.88, respectively) (Figure 2, right column of panels).

## Discussion

Extensive inherited and acquired mutations characterize HAS1 in patients with B-cell malignancies [8]. *HAS1* overexpression has also been reported for some solid tumors, including prostate, ovarian and bladder cancers [1]; [12]–[14]. To avoid the uncertainties inherent in genome-wide association studies, we sought susceptibility polymorphisms in a candidate gene, *HAS1*, based on the biology of the gene in cancer patients. By sequencing in 832 patients and 582 healthy controls (a total of 1,414 individuals) a segment of the *HAS1* gene known to influence aberrant HAS1 splicing [7]; [8], we show that the presence of *HAS1* SNPs is strongly associated with the diagnosis of a systemic B-cell malignancy (range of p = 0.005 to p = 10^−5^, OR = 1.5 ranging to OR = 2.1, overall risk = 1.78), but not with a solid tumor. For the Icelandic family members the overall risk was 5.8; no such risk was seen for breast cancer or for the cohort of patients with sporadic MGUS that have no known familial predisposition. The observation that HAS1 intronic SNPs do not confer risk to patients with sporadic MGUS likely reflects the fact that only a very small proportion are at risk of transformation to frank malignant disease. It seems likely from the Icelandic familial analysis that if biomarkers were available that preferentially select for only those MGUS that will transform to frank malignancy, a risk association would become detectable. In the Icelandic family kindred, *HAS1* SNPs were more frequent in affected members (those with monoclonal gammopathies or hype-responder phenotype) than in unaffected family members (p = .0005), confirming the association with risk in a four generation family having a shared genetic heritage.

The increased risk associated with minor allele genotypes in *HAS1* intron 3, as first shown here, is supported by our previous work [1]; [8]; [15]. In some blood cancer patients, *HAS1* is a hypermutated gene [8] that undergoes aberrant splicing to generate splice variants that correlate with poor survival [4]. *In vitro* mutagenesis of *HAS1* intron3 alters *HAS1* splicing patterns, causing transfectants to acquire the splicing pattern seen in MM patients[7]; [8]. *In silico* and *in vitro* mutagenesis analyses predict that *HAS1* intron3 plays a central role in clinically relevant splice site selection and consequent aberrant intronic splicing of *HAS1* [7]; [8]. Intronic SNPs can have a functional impact by affecting gene regulation and splicing mechanisms [16]–[18], confirming that intronic SNPs have a potentially strong influence on oncogenic events. In MM and WM, *HAS1* genetic variations are distributed in or near splicing elements [8]; [15] and can direct aberrant splicing of *HAS1* [7]; [8]. In transfected cells, the properties of aberrant HAS1 variants are dominant over those of the normally expressed HAS1 full length form [6].

The independent Icelandic kindred is a small but relatively homogeneous population with a common ancestry. Affected family members exhibit hyper-Ig synthesis *in vitro* and some have MGUS, WM or MM. The linked *HAS1* intron3 SNP alleles and genotypes of affected members, but not an exon3 SNP, were significantly different from those of unaffected members (genotype frequencies p = 0.007, allele frequencies p = 0.0005).

In the pooled analysis of SNP genotypes for all patient and control groups, the odds ratio for B cell malignancy (OR_trend_) was 1.78 and for affected Icelandic family members, OR was 5.8. These odds ratios, especially when considered in the context of the familial analysis, suggest minimal or no influence of cryptic population stratification. Risk predictions may be directly correlated with the identified SNPs, or may result from unknown mutation(s) outside the sequenced region but in linkage disequilibrium with the detected SNPs. The OR for breast cancer patients and MGUS did not differ from unity, suggesting that the increased risk conferred by HAS1 SNPs is selective for systemic B-cell malignancies.

Family members of patients with MGUS or MM have a two-three-fold higher risk of developing MGUS and multiple myeloma [19]–[22]. CLL and WM include both sporadic and familial forms of the disease [23]–[26]. Inherited polymorphisms are associated with both CLL and WM [15]; [25]; [27]–[31], and a strongly recurrent somatic mutation with WM [32]. The odds ratios found here for CLL and WM were considerably more pronounced than those found for MM which appears to have less familial influence. However, this work demonstrates for the first time that an increased frequency of *HAS1* intron3 SNPs is common among all three systemic B-cell malignancies, possibly indicating shared genetic predispositions that culminate in malignant transformation of B-cells.

We employed sequencing for this study because it provides for unequivocal allele calling in each patient, as well as identification of any closely linked rare mutations that might influence risk. An advantage in SNP detection by direct DNA sequencing is the complete information it yields, including haplotype relationships. Recent literature supports our approach [33] and emphasizes the use of sequencing to detect direct associations between disease and casual SNPs [34]. Our analysis was restricted to polymorphisms in *HAS1* exon3 and intron3, a mutation-rich region that is involved in aberrant splicing of *HAS1* exons and introns [7]; [8]. Our work suggests that the three linked *HAS1* intronic SNPs may predispose to aberrant splicing, or are in linkage disequilibrium with a causative functional variant located outside exon3 and intron3 of HAS1. This study provides strong evidence to support the hypothesis that *HAS1* contributes to a genetic risk of B-cell malignancy.

In conclusion, our findings suggest that genetic variants in *HAS1* are associated with risk of B-cell malignancy in MM, CLL and WM. Because the *HAS1* intron3 SNPs are present at significantly higher frequencies in individuals who have developed a systemic B-cell cancer, these results suggest that intronic *HAS1* SNPs predispose to systemic B-cell cancers and as inherited characteristics may thus act at an early stage of oncogenesis. Although multiple genes are certainly involved, this work supports the idea that *HAS1* intron3 SNPs have a strong impact on molecular events, particularly on aberrant HAS1 pre-mRNA splicing events that contribute to the malignant phenotype in B-cell cancers. Molecular therapy to target this region of *HAS1* warrants evaluation.

## Supporting Information

File S1 **Suppporting Tables.** Table 1, Primer sequences used for PCR or sequencing reactions. Table S2, Components of PCR mixture. Table S3, HAS1 SNPs observed in this study.
(DOCX)
