# Supplementary material for: Inherited Polymorphisms in Hyaluronan Synthase 1 Predict Risk of Systemic B-Cell Malignancies but Not of Breast Cancer
Source: PLoS One. 2014 Jun 20;9(6):e100691. doi: 10.1371/journal.pone.0100691 (PMC4065063; doi:10.1371/journal.pone.0100691)
Supplement: File S1 — Suppporting Tables. Table 1, Primer sequences used for PCR or sequencing reactions. Table S2, Components of PCR mixture. Table S3, HAS1 SNPs observed in this study. (DOCX) [file pone.0100691.s001.docx]

**File S1: Supplementary Information**

**Table of Contents**

**Supplementary Table 1.** Primer sequences used for PCR or sequencing reactions

**Supplementary Table 2.**  Components of PCR mixture

**Supplementary Table 3.** HAS1 SNPs observed in this study

Table S1. The primer sequences used for PCR or sequencing reactions

| ***HAS1 Primers*** | ***Sequence 5'-3'*** |
| --- | --- |
| 5' exon3 | GGGGTCTGTGCTGATCCTGG |
| 3' intron3 | AACTGCTGCAAGAGGTTATTCC |
| 3' exon 4 | CATGCACACACGCTAGGATA |
| M13R | CAGGAAACAGCTATGAC |
| T7 | TAATACGACTCACTATAGGG |

**Table S2: Components of PCR mixture**

| **Components** | **Volume** | **Final Concentration in**  **50 μl reaction mixture** |
| --- | --- | --- |
| 10X Buffer | 5 μl | 1 X |
| MgSO_4_ | 2 μl | 2mM |
| dNTPs | 1 μl | 0.4μM |
| Forward primer | 1 μl | 0.4μM |
| Reverse primer | 1 μl | 0.2mM |
| Template | Varies | 50 ng |
| HiFi taq | 0.2 μl | 0.5U |
| Sigma H_2_O | Make up to 50 μl |  |

**Table S3. HAS1 SNPs observed in this study**

| **SNP number** | **Location** | **frequency** |
| --- | --- | --- |
| rs61736495 | Exon 3 | 0.03 |
| rs78761398 | Exon 3 | 0.01 |
| rs75142551 | Exon 3 | 0.01 |
| rs11084111 | Exon 3 | 0.20 |
| rs11152339 | Intron3 | 0.02 |
| rs11084110 | Intron3 | 0.7 |
| rs11084109 | Intron3 | 0.7 |
| rs11669079 | Intron3 | 0.7 |
| rs80094546 | Intron3 | 0.03 |
| rs58381226 | Intron3 | 0.03 |
